# Greater mechanistic understanding of the cutaneous pathogenesis of Stevens–Johnson syndrome/toxic epidermal necrolysis can shed light on novel therapeutic strategies: a comprehensive review

**DOI:** 10.1097/ACI.0000000000000993

**Published:** 2024-05-17

**Authors:** Emeka D. Ogiji, Nourah Aboheimed, Kehinde Ross, Calum Voller, Ryan Siner, Rebecca L. Jensen, Carol E. Jolly, Daniel F. Carr

**Affiliations:** a Department of Pharmacology and Therapeutics, University of Liverpool, Liverpool, UK; b Department of Pharmacology and Therapeutics, Ebonyi State University, Abakaliki, Nigeria; c Department of Pharmacy Practice, Princess Nourah bint Abdulrahman University, Saudi Arabia; d School of Pharmacy and Biomolecular Sciences, Liverpool John Moores University; e School of Medicine, University of Liverpool, Liverpool, UK

**Keywords:** drug hypersensitivity, drug repurposing, pathogenesis, Stevens–Johnson syndrome, toxic epidermal necrolysis

## Abstract

**Purpose of review:**

Stevens–Johnson syndrome/toxic epidermal necrolysis (SJS/TEN) are severe cutaneous adverse drug reactions (SCARs) characterized by widespread epithelial detachment and blistering, which affects the skin and mucocutaneous membranes. To date, therapeutic interventions for SJS/TEN have focused on systematic suppression of the inflammatory response using high-dose corticosteroids or intravenous immunoglobulin G (IgG), for example. No targeted therapies for SJS/TEN currently exist.

**Recent findings:**

Though our understanding of the pathogenesis of SJS/TEN has advanced from both an immunological and dermatological perspective, this knowledge is yet to translate into the development of new targeted therapies.

**Summary:**

Greater mechanistic insight into SJS/TEN would potentially unlock new opportunities for identifying or repurposing targeted therapies to limit or even prevent epidermal injury and blistering.

## INTRODUCTION

Stevens–Johnson syndrome (SJS) and toxic epidermal necrolysis (TEN) are severe skin blistering adverse drug reactions (ADRs) characterized by widespread keratinocyte death and epidermal detachment [1]. Reactions are immune-mediated, type IV (delayed onset) reactions that are CD8^+^ T-cell-driven. SJS/TEN has an estimated overall incidence of 5.76 cases per million person-years [2] and a mortality rate of 5–25% [3]. They are most caused by antiepileptics [4], antiuricemics [5] and antibiotics [6] but have been observed secondary to over 250 licensed small molecules and biologics [7]. Whilst patients with SJS/TEN are often managed in ITUs, the skin detachment in TEN can be so severe (>30% body surface area) that it requires treatment in specialist burns units [8]. The mean hospital stay for an SJS patient is 7.0 days with 1.7 days in an intensive treatment unit (ITU), rising to 12.6 days (4.9 in ITU) for TEN [9].

A number of guidelines for the clinical management of SJS/TEN have been devised [10–12]. There remains, however, little consensus on the most efficacious treatment regimen. Evidence advocates the use of corticosteroids [13], cyclosporine [14], plasmapheresis [15^▪^], immunoglobulins [15^▪^]. But these drugs have diverse and nonspecific biological effects on the systemic inflammatory response in SJS/TEN, relying on generalized immune suppression, with evidence suggesting little difference in clinical outcomes between the different therapies [16].

The use of TNF-α inhibitors, such as etanercept, has emerged as an effective treatment of SJS/TEN, with reports of rapid skin re-epithelialization after use [17]. A recent Cochrane review highlights anti-TNF therapies as having the most compelling supportive evidence for efficacy in SJS/TEN [18^▪▪^]. Currently, however, there is limited clinical trial evidence to guide practice and, to date, little is understood about the downstream effect of TNF-α in SJS/TEN and how it is modulated by anti-TNFs. Lack of mechanistic understanding means patients are receiving additional immunosuppression whilst others remain on oscillating doses of corticosteroid. This is not an optimal clinical position, but in the absence of further mechanistic understanding providing an evidence base for therapeutic advancement, this will likely remain the status quo. One thing is clear: there are currently no therapies targeting the pathogenic mechanisms of epidermal detachment in SJS/TEN in use.

The ultimate ambition should be the development or repurposing of therapeutics, which can be administered at the earliest indication of cutaneous SJS/TEN symptoms to prevent/limit epidermal detachment. This could also be used alongside systemically administered immune modulators wherever required, though the aim should be targeted therapy over nontargeted systemic treatments.

This review discusses how greater understanding of mechanistic biomarkers and pathogenesis could inform the discovery of novel targeted therapeutic interventions and repurposing of existing agents for the treatment of SJS/TEN, and highlights some key examples. 

**Box 1:**
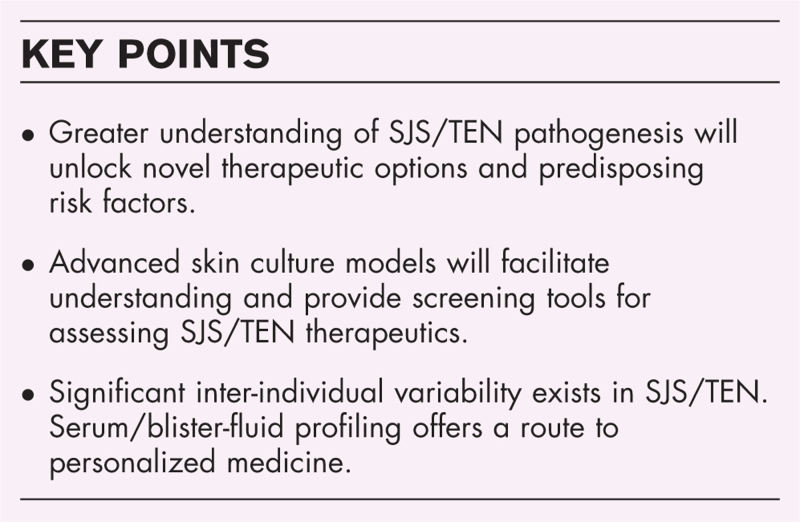
no caption available

## MECHANISTIC INSIGHT INTO STEVENS–JOHNSON SYNDROME/TOXIC EPIDERMAL NECROLYSIS PATHOGENESIS

Significant advances have been made in our understanding of the immunological mechanisms behind SJS/TEN, but gaps in our knowledge, particularly around the pathogenesis of keratinocyte death and epidermal detachment still exist. Greater insight into these aspects could yield significant advances in targeted SJS/TEN therapies where none currently exist.

The proposed downstream effect of many molecules implicated in SJS/TEN is the induction of keratinocyte death. Indeed, apoptotic cell death has been proposed to have a role in the pathogenesis of SJS/TEN [19,20], with both the extrinsic and intrinsic pathways leading to caspase-3 activation and keratinocyte death. The extrinsic pathway utilizes TNF-α, Fas/FasL and TNF-related apoptosis-inducing ligands, to bind cell surface death receptors and activate caspase-8, which in turn activates caspase-3 associated cell death. In the intrinsic pathway, CD8^+^ T-cell induced cellular stress leads to the release of BAX/BAK, stimulating mitochondrial release of cytochrome c, causing the inactive procaspase-9 cleavage and caspase-3 activation. Caspase-3 can also be activated by granulysin, perforin, granzyme B and Fas-ligand released by CD8^+^ T cells, Natural Killer (NK) cells and macrophages [19–21].

Necroptotic cell death also contributes to skin toxicity in SJS/TEN. During the early phases of the disease, skin-infiltrating CD8^+^ T cells trigger the production of lipocalin-2, leading to the formation of neutrophil extracellular traps (NETs) via NETosis [22]. During this process, LL-37 is released from neutrophils causing keratinocytes to express formyl peptide receptor 1 (FPR1). Released FPR1 makes the keratinocytes vulnerable to necroptosis and creates a feedback loop leading to further production of LL-37 and an amplification of necroptosis [22]. The potential to repurpose compounds to modulate this mechanism is discussed in detail as follows. Interaction between annexin A1 and FRP1 [23] is thought to be a trigger for necroptosis. In SJS/TEN causal drug exposure causes monocytes to secrete annexin A1, which binds FRP1 on the keratinocyte cell surface leading to further FRP1 expression [23] and downstream necroptosis. Additionally, it has also been suggested that necroptosis can occur when annexin A1 is up-regulated [23]. Thus, inhibitory molecules targeting annexin A1, such as AC-2-26, may represent a tool to modulate necroptosis in SJS/TEN [24].

A number of immunomodulatory proteins have been observed as elevated in SJS/TEN patient serum and/or blister fluid during the acute phase of the reaction, suggesting a putative role in the pathogenesis of skin manifestations of SJS/TEN (Table 1). These include pro-inflammatory cytokines [e.g. TNF-α, interleukin 1 (IL-1), and interferon-γ (INF-γ)], and soluble cytolytic proteins (perforin [25,26], granzyme B [26], and granulysin [27,28]). Additionally, chemokine receptors/ligands [CC motif chemokine ligand 27 (CCL27), cutaneous T-cell-attracting chemokine (CTACK), CC motif chemokine receptor 6 (CCR6) and CCR10] have been shown to be overexpressed in SJS/TEN, suggesting they may also play a part in the pathogenesis [29]. Although research, in most cases, is limited to their roles as biomarkers of SJS/TEN, many have functions, which are likely to contribute to cutaneous pathogenesis (Table 1). Modulation of these proteins using existing approved therapeutics may offer novel repurposing opportunities in SJS/TEN. Indeed, limited clinical assessment of TNF-α inhibitors etanercept and infliximab, and IL-5 inhibitor benralizumab in SJS/TEN, has already been undertaken suggesting they may be viable therapeutic options [30^▪^,^31^,32^▪^].

**Table 1:** Immune cell-derived pro-inflammatory cytokines, chemokines and cytolytic proteins involved in the cutaneous pathogenesis of Stevens–Johnson syndrome/toxic epidermal necrolysis

SJS/TEN-associated molecules	Cellular source	Significance in SJS/TEN	Proposed role in SJS/TEN pathogenesis	Potential targeted therapies (current indication/trial)	Clinically assessed in SJS/TEN?
CCL27/CCR10	Keratinocytes/skin-homing T cells, fibroblasts, endothelial cells	Elevated in serum [39]. Up-regulated in keratinocytes and epidermal suprabasal layer [29]. Upregulated mRNA expression SJS/TEN patient PBMCs [29]	T-cell recruitment to inflamed skin [40]	BI-6901 (CCR10 inhibitor for contact dermatitis (*in vivo*)) [41]	No
CXCL9	Keratinocytes, macrophages	Elevated in serum [42]	Stimulation of T-cell cytokine production and promotion of Th1 cell proliferation	–	
CXCL10	Keratinocytes, macrophages	Elevated in serum [42]	Modulates T-cell migration to lesional epidermis [43]	Atorvastatin [44]	No
Granulysin	CD8+ T cells NK cells	Elevated in serum/blister fluid [27]	Induces keratinocyte apoptosis [27]	–	–
Granzyme B	CD8+ T cells NK cells	Elevated in serum [26]	Induces keratinocyte apoptosis [45,46]	–	–
LL-37	Neutrophils Keratinocytes	Elevated in serum [22]	Induces keratinocyte necroptosis [22]	Glycoaminoglycans [47]. GM-1111 [48]	No
Perforin	CD8+ T cells	Elevated in serum, related to disease severity [26]	Induces keratinocyte apoptosis [49]	SN34960 (experimental perforin inhibitor) [50]	No
IFN-γ	Th1 cells CD8+ T cells NK cells	Elevated in serum [42]	Modulates T-cell migration to lesional epidermis	–	–
IL-2	Activated CD4+ and CD8+ T cells	Elevated in serum [51]	Stimulates T-effector cell activation and regulation	Cyclosporine [52]	Yes [53] [Meta-analysis (*n* = 358)]
IL-5	Eosinophils, CD4+ and CD8+ T cells	Elevated secretion from SJS/TEN patient drug-exposed PBMCs [54]	Eosinophil differentiation [45,46]	Benralizumab	Case report (*n* = 1) [32^▪^]
IL-6	Macrophages T cells Keratinocytes	Elevated in serum [33] and blister fluid [55]. Correlates with skin re-epithelialization [56]	T-cell activation and proliferation. Production of CD8+ T cells [56]	Tocilizumab (rheumatoid arthritis). Sarilumab (rheumatoid arthritis)	No No
IL-12	Th1 cells Dendritic cells		Elevated in serum [57]	Enhances activity of NK cells [58] and CD8+ T cells [59]	Ustekinumab (ulcerative colitis) [60]
IL-13/IL-4	Th2 cells	Elevated in plasma, skin [61] and serum [62]	Dendritic cell induction of Th2 differentiation [63]	Lebrikizumab (atopic dermatitis) [64]. Dupilumab (atopic dermatitis) [65]	No No
IL-15	Regulatory T cells (Treg)	Elevated in serum, correlates with severity/mortality [33,34]. Increased skin transcript levels [34]	Development and homeostasis of NK cells and CD8+ T cells [66]	Hu-Mik Beta-1 (T-cell large granular lymphocytic (T-LGL) leukaemia) [67]	No
IL-17	Th17 cells	Elevated in serum, correlates with severity [68]	Neutrophil mobilization and activation [69]	Brodalumab (psoriasis) [70] Bimekizumab, secukinumab (plaque psoriasis) [71,72]	No No
IL-33	Keratinocytes	Elevated in serum [73]	Enhances the production of Th2 cytokines (IL-5 and IL-13) [74].	ZINC59514725 (experimental IL-33 inhibitor) [75]	No
Soluble FasL	CD8+ T cells NK cells	Elevated in serum [57,76]	Induces keratinocyte apoptosis [77]	–	–
TARC	Th2 cells	Elevated in serum [57]	–	–	–
TNF-α	CD8+ T cells NK cells	Elevated in serum [39] and blister fluid [55]	Induces keratinocyte apoptosis/necroptosis [78^▪^]	Etanercept Infliximab	RCT (*n* = 96) [31] Cochrane Review (*n* = 308) [18^▪▪^] Meta-analysis (*n* = 31) [30^▪^]

Increasing evidence is emerging of the pleiotropic effects of interleukin 15 (IL-15) in the pathogenesis of SJS/TEN. Serum IL-15 levels have been demonstrated to correlate closely with severity and mortality, and to enhance the cytotoxicity of NK cells in TEN [33]. Furthermore, studies of SJS/TEN skin have suggested significant up-regulation of the receptor, IL-15Rα, through which IL-15 elicits its effects [34]. For this reason, there is currently much focus on novel therapies targeting IL-15-mediated pathways, in particular Janus kinase/signal transducers and activators of transcription (JAK/JAK-STAT) [35]. A number of JAK inhibitors are already licensed (ruxolitinib), or in development (tofacitinib) for use in other inflammatory skin conditions [36] including psoriasis [37] and atopic dermatitis [38]. As such, JAK inhibitors offer promise in the treatment of SJS/TEN. It is not currently clear at this moment in time whether pan-JAK inhibitors, such as tofacitinib would be more efficacious than specific JAK inhibitors in SJS/TEN but work is ongoing to assess this.

Using appropriate in-vitro, ex-vivo and in-vivo models, these markers can be evaluated as targets for the development of novel therapeutic agents for SJS/TEN.

## MECHANISTIC BIOMARKERS OF STEVENS–JOHNSON SYNDROME/TOXIC EPIDERMAL NECROLYSIS AS NOVEL THERAPEUTIC TARGETS

A number of circulatory and tissue-specific biomarkers of cell/tissue injury in SJS/TEN have been reported, with many produced as a consequence of the pathogenesis. However, a number is also thought to contribute to or exacerbate the damage process.

As an example, high-mobility group box 1 (HMGB1), a member of the damage-associated molecular pattern (DAMP) family, is actively released in its acetylated form by stimulated macrophages [79,80] and passively released, in its nonacetylated form, by dying cells [81,82]. As such, HMGB1 captures two key events in SJS/TEN pathogenesis during the abnormal immune response and the subsequent cell death. Indeed, levels of HMGB1 have been shown to increase in SJS/TEN patient sera and/or blister fluid [83–85] and decrease in skin [78^▪^,^86^]. Extracellular HMGB1 can be a potent chemoattractant for neutrophils, acting as a strong DAMP signal to stimulate cytokine production, and potentially serving as an alarm signal within the T-cell activation cascade [82,87].

HMGB1's immunological effect depends on posttranslational redox state of three cysteines at positions 23, 45 and 106 [82,88], which regulate its receptor binding. A thiol group on HMGB1 cysteine 106 (disulphide redox form), is essential for both TLR4/MD2 receptor complex binding and TNF-α production [87]. While the fully reduced HMGB1 allows the formation of a heterocomplex with chemokine CXCL12 which then binds the CXCR4 receptor [89].

The mechanistic role of HMGB1 in SJS/TEN remains unclear, although studies in other inflammatory and immune-mediated conditions (sepsis [90], rheumatoid arthritis [91] and psoriasis vulgaris [92]) hint at a possible pathological role. HMGB1 can alter the immune environment and modulate Treg/Th17 ratio by enhancing Th17 activation to increase IL-17 production [93,94]. In addition, HMGB1 redox forms have been found to down-regulate immune checkpoints (notably CTLA-4) on Tregs dampening immunosuppressor mechanisms [95]. The ability to alter the immune response via cytokine production, immune cell recruitment suggests HMGB1 inhibition, with demonstrably well tolerated compounds such glycyrrhizin [96], could be a valid therapeutic target for the prevention of further inflammation and tissue damage in SJS/TEN.

## DRUG REPURPOSING

Recent developments in our understanding of SJS/TEN pathogenesis have highlighted some interesting opportunities for potential drug repurposing:

### Matrix metalloproteinase inhibitors

**FIGURE 1:**
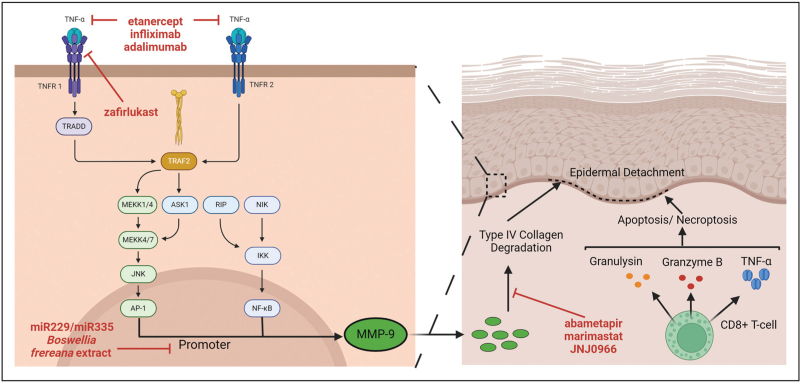
Putative therapeutic interventions of the proposed molecular mechanisms of TNF-α-induced, MMP9-mediated, epidermal detachment in Stevens–Johnson syndrome/toxic epidermal necrolysis. Small molecules which modulate TNF-α activity, MMP-9 transcription/expression and activity are highlighted in red. Created with BioRender.com.

Matrix metalloproteinase 9 (MMP-9), is overexpressed in the skin of SJS/TEN patients, suggesting a role in the observed epidermal detachment [97,98,99^▪^]. A recent study suggests that elevated MMP-9 expression in the epidermis, with the subsequent increased collagenase activity are proposed as a pathogenic mechanism, underlying the epidermal detachment seen in SJS/TEN [99^▪^]. The study also suggests that these effects are TNF-α-dependent and can be mitigated by the TNF inhibitor etanercept. Other MMP-9 antagonists, for example, abemetapir [100], andecaliximab [101] and *Boswellia frereana*[102] extract, could also be potentially repurposed for SJS/TEN management. The theoretical pathogenic pathway of TNF-α-induced MMP9-mediated epidermal detachment and examples of how it could be therapeutically modulated are shown in Fig. 1.

In addition to small molecule drugs for MMP9 modulation, alternative modalities such as MMP-9-regulating microRNAs (miRNAs) also offer intriguing possibilities. Mimics of miR-229 and miR-335 have been shown to enhance wound recovery in mouse models of diabetic healing by down-regulating MMP-9 expression [103]. Application of nanoparticles delivering MMP-9 inhibitory peptidomimetics to affected areas [104], may also offer effective potential anti-MMP-9 therapies.

### LL-37 and glycosamnioglycans

A role for NETosis in SJS/TEN is evidenced by early NET accumulation in lesional and perilesional skin, and by the overexpression of LL-37, an antimicrobial peptide of the cathelicidin family, in serum and blister fluid, which is not seen in the other SCARs and blistering disorders [22].

The release of LL-37 both augments NETosis and induces the necroptotic FPR1-annexin A1 axis in keratinocytes, eliciting further release of LL-37-initiating necroptosis in adjacent cells, exacerbating the injury. Aberrant NET formation has been implicated in the pathogenesis of a number of autoimmune conditions (rheumatoid arthritis [105] and lupus [106]), through the activation of inflammasomes by LL-37 and the generation of autoantigens such as those against the NET-released dsDNA [107].

Indeed, Kinoshita *et al.* show that, in neutrophils derived from healthy volunteers, only SJS/TEN sera and blister fluid, and not that of heathy controls, triggers NETosis with the ensuing LL-37 release [43]. As such, LL-37-targeting therapies may hold promise in their ability to terminate the abnormal positive feedback loops.

Glycosaminoglycans (GAGs), including the endogenous skin components dermatan and hyaluronic acid, are anionic polysaccharides with often promiscuous activity due to variable sulphation [108]. The cationic nature of LL-37 renders its antibacterial activity susceptible to inhibition by the anionic GAGs [109], raising the possibility that their presence, endogenously or exogenously, may moderate the pathogenesis of SJS/TEN. Indeed, lower levels of endogenous cutaneous GAGs may represent a potential risk factor for developing the more severe SJS/TEN, rather than milder self-resolving maculopapular exanthema during cutaneous drug hypersensitivity reactions. However, further investigation of inter-individual variability in endogenous cutaneous GAGs and their correlation with ADR risk is required.

## DEVELOPMENT OF IN-VITRO/EX-VIVO MODELS OF STEVENS–JOHNSON SYNDROME/TOXIC EPIDERMAL NECROLYSIS

Our ability to identify and evaluate new SJS/TEN therapeutic options is dependent on having appropriate experimental tools at our disposal. There is currently a lack of physiologically relevant in-vitro, ex-vivo and in-vivo skin models applied to understanding of the molecular pathogenesis of SJS/TEN. Such models could help, not only to characterize the molecular and cellular basis of pathogenesis but also be utilized for diagnostic and drug screening applications.

Traditional 2D skin models typically incorporate primary human or immortalized keratinocytes (such as HaCaTs), either in isolation, or co-cultured with other relevant cell types, (e.g. monocytes [110], or fibroblasts [111]). Such 2D models have been previously utilized to elucidate keratinocyte toxicity to molecules implicated in SJS/TEN pathogenesis, such as granulysin [26], TNF-α [78^▪^] and LL-37 [22]. However, in the context of SJS/TEN, such models are limited in their capability to recapitulate the complexities of the signalling between the many different cell types of the skin and the pathogenesis of epidermal detachment [112].

Bioengineered human skin equivalents (HSEs) are 3D skin models, which are composed of primary human cells (keratinocytes, fibroblasts and/or stem cells) and components of the extracellular matrix (ECM) [113]. Two different HSEs are used for research purposes: epidermal-only equivalents or reconstructed human epidermis (RHE) and full thickness skin equivalents with both epidermal and dermal compartments.

HSEs have been used for a wide range of research, including psoriasis studies [113,114], and could feasibly be applied to SJS/TEN. Like SJS/TEN, the pathophysiology of psoriasis involves an interaction between keratinocytes and immune cells, which can be recapitulated *in vitro* using 3D skin models with T cells incorporated between the dermal and epidermal layers, mimicking the skin phenotype and cytokine/transcription factor profiles [115].

The advantage of HSEs over 2D models the ability to recapitulate epidermal/dermal detachment at the basement membrane [116], a key feature of SJS/TEN. In addition, HSEs offer batch consistency and absence of significant variability in morphology. This is optimal for drug screening applications, for example. Indeed, HSEs have been used to identify therapies for psoriasis, supporting its utility for use in drug development [117]. These models can also be scaled up for compound screening using Alvetex scaffold 12-well inserts as exemplified in a melanoma research [118].

Despite the significant advances in skin tissue equivalents in recent years, the most appropriate model of skin remains fresh biopsies. However, obtaining fresh skin from SJS/TEN patients in significant numbers, especially at the time of reaction, is logistically challenging given the rarity of such reactions. Encouragingly, recent studies have shown that an SJS/TEN phenotype can be induced in healthy skin biopsies using sera taken from SJS/TEN individuals taken at time of reaction [78^▪^,99^▪^]. This has the potential to allow mechanistic investigation of SJS/TEN pathogenesis in a model, which is significantly more accessible to researchers. In future, it may be possible to produce skin explant/immune cell co-culture models, which are an accurate recapitulation of the clinical phenotype and could be utilized as a prognostic/causality tool of for drug screening.

## IN-VIVO MODELS FOR PRECLINICAL EVALUATION

Animal models are a crucial step in evaluating efficacy and translation of novel therapeutics in SJS/TEN. Previously, SJS/TEN patient peripheral blood mononuclear cells (PBMCs) have been intravenously injected into mice, which are then pulsed with causal agents [119]. This model, however, has limited utility for understanding skin injury and perturbation in SJS/TEN as symptoms were limited to ocular toxicity. However, an epidermal-specific inhibitor of apoptosis protein (IAP)-deficient mouse model, which exhibits a TEN-like phenotype [120] has been reported, which has huge potential as a tool for the evaluation of SJS/TEN therapies and for deeper understanding of pathogenic mechanisms, particularly those mediated by TNF-α. In-vitro studies in keratinocyte cell lines have suggested that chemical inhibition of IAPs (using the SMAC inhibitor BV-6) [78^▪^] sensitizes them to TNF-α-induced toxicity. Variability in cutaneous IAP expression and thus TNF-α sensitivity could explain variability in severity if drug hypersensitivity phenotype. Furthermore, modulation of IAP expression could represent an additional putative therapeutic target.

## PERSONALIZED TREATMENT REGIMENS FOR STEVENS–JOHNSON SYNDROME/TOXIC EPIDERMAL NECROLYSIS

Whilst many different novel therapeutic concepts could be considered for SJS/TEN, moving forward we need to consider the application of personalized treatment regimens. Owing to the heterogeneity SJS/TEN of patients, the current standard of care, (e.g. corticosteroids) are only effective in a subset of individuals. It is likely that keratinocyte death may be facilitated by all or some of the immunopathological mediators described above to differing degrees in different patients. This may account for the variation in the efficacy for some immunomodulatory treatments.

**FIGURE 2:**
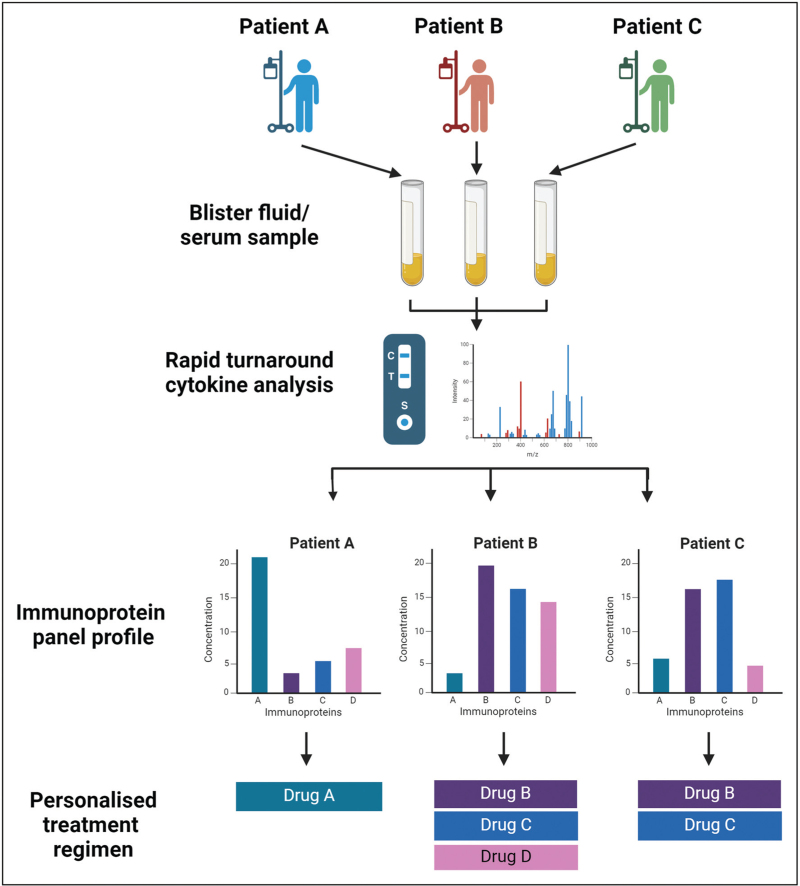
Hypothetical process for a personalized therapeutic approach for the treatment of acute Stevens–Johnson syndrome/toxic epidermal necrolysis. Patient serum and/or blister fluid would be sampled and a quick turnaround assay would provide a detailed assessment of a panel of key immunoproteins (cytokines, chemokines and soluble cytotoxic proteins) implicated in SJS/TEN pathogenesis. The panel would then inform a personalized drug regimen which takes into consideration the heterogeneity of the immune response between SJS/TEN patients. Created with BioRender.com.

We now have a significant body of evidence for the role of cytokines, chemokines and soluble cytotoxic proteins in SJS/TEN (Table 1). It is entirely plausible that we could utilize data on the levels/presence of these markers in patient sera and/or blister fluid, to identify the key protagonists in any given individual and deliver a truly personalized regimen of therapeutics (Fig. 2). Given the acute onset of SJS/TEN, quantification/detection of cytokines and other factors prior to treatment would require a fast and robust assay, for example, a lateral flow test. Indeed, studies have already described such a test for interleukin 6 [121], which could be adapted to other serum and blister fluid cytokines.

The feasibility of such an approach to personalizing SJS/TEN treatment is clearly dependent on the future development of technologies to form a rapid panel-based test. However, its application could have significant benefits in improving patient outcomes and reducing the adverse effects and variable efficacy of the current systemic therapeutic options.

## TRANSLATION FROM BENCH TO BEDSIDE: THE CHALLENGES

SJS/TEN is rare and this creates challenges for the generation of evidence-based treatment, because of a lack of numbers for both laboratory research and clinical trials [122]. Accessing patients in a timely manner particularly for acute reaction sampling, can be challenging because of logistical limitations. There is a need for connected clinical and research resources, including centralized treatment models and patient registers, which could greatly enhance research and treatment outcomes in SJS/TEN.

The translation of experimental findings for novel SJS/TEN therapeutics into clinical application represents a significant problem. Repurposing of existing drugs used to treat other inflammatory conditions looks to be the most realistic way forward in the short-term, where the requirement for preclinical evaluation is largely negated. Indeed, the case of TNF-α inhibitor etanercept clinical implementation demonstrates the effectiveness of this methodology [31]. There is, however, a demonstrable need to establish the effectiveness of therapeutic strategies which target the nonimmune-mediated mechanisms of pathogenesis in SJS/TEN, which will require significant ambition to achieve.

## CONCLUSION

Understanding of the immunological component of SJS/TEN pathogenesis has advanced at pace in recent years and provided us with mechanistic biomarkers which may yet yield targeted therapies. However, a deeper understanding of the pathogenesis of keratinocyte injury and epidermal detachment has the potential to aid the discovery of further novel or repurposed therapies for SJS/TEN. Furthermore, given the complexity of SJS/TEN and patient heterogeneity, a personalized approach to targeted therapies should also be considered in order to optimize short-term and long-term clinical outcomes. In doing so, we will be able to better equip physicians with both the information and tools to treat these often-life-threatening reactions.

## Acknowledgements

*None.*

### Financial support and sponsorship

*D.C. has received research funding from ViiV Healthcare and Roche which is unrelated to this article. No other authors report any financial support.*

### Conflicts of interest

*There are no conflicts of interest.*

## REFERENCES AND RECOMMENDED READING

Papers of particular interest, published within the annual period of review, have been highlighted as:

▪ of special interest
▪▪ of outstanding interest
